# Clinical Evaluation of a Novel, Chemically-Generated, Single-Use Negative Pressure Wound Therapy System for the Management of Closed Surgical Incisions

**DOI:** 10.7759/cureus.86148

**Published:** 2025-06-16

**Authors:** Jon A Mathy, Alpesh U.C. Patel, John S Buan, Jeffrey Ustin, Robin Martin

**Affiliations:** 1 Plastic and Reconstructive Surgery Department, Middlemore Hospital, Auckland, NZL; 2 Department of Surgery, School of Translational Medicine, Monash University, Melbourne, AUS; 3 Orthopaedic Department, Middlemore Hospital, Auckland, NZL; 4 Chief Technical Officer, Aatru Medical, LLC, Cleveland, USA; 5 Owner, Buan Consulting Solutions, LLC, Minneapolis, USA; 6 Medical Director, Aatru Medical, LLC, Cleveland, USA; 7 Trauma, UH Cleveland Medical Center, Cleveland, USA; 8 Freelance Scientific Consultant, Robin Martin PhD Scientific Consulting, Selby, GBR

**Keywords:** chemically generated negative pressure, closed incision negative pressure wound therapy, non-electrical negative pressure wound therapy device, oxygen reduction technology, surgical site infection

## Abstract

Background

Closed incision negative pressure wound therapy (ciNPWT) is a successful strategy to improve surgical outcomes. However, its use can be limited by the cost of electrically-powered ciNPWT systems, especially in developing countries. A novel negative pressure wound therapy (NPWT) device that utilizes a chemical reaction to create negative pressure promises to be significantly less costly.

Method

A first-in-human clinical study was completed to evaluate the safety and performance of a novel ciNPWT device (NCT04488666). The primary endpoint was the longevity of the delivery of negative pressure to the wound over seven days. Wound healing was assessed on day seven and day 30. Assessment of the scar quality on day 14 and day 30, exudate management, and ease of use were also undertaken.

Results

A total of 23 patients were enrolled in the study (mean age 65.0±11.9 years; mean BMI 30.9±6.8 kg/m^2^ with a mean wound length of 7.6±2.6 cm, predominantly in elective cutaneous surgery and spinal surgery procedures. The new device was used to maintain negative pressure and manage wound exudate for a mean of 6.3±1.6 days per incision. The mean number of devices used per incision was 1.2±0.5. The Additional treatment; Serous discharge; Erythema; Purulent exudate; Separation of deep tissues; Isolation of bacteria; and Stay as inpatient for a prolonged period (over 14 days) or ASEPSIS and the Patient and Observer Scar Assessment Scale (POSAS) scores indicated overall normal healing and scar quality. There were a total of nine adverse events in five patients. Wounds treated with the new device were not painful. Participant and clinician assessments showed that the devices were easy to use in the hospital and easy to manage at home with routine activities. The device made no audible sound during its use.

Conclusion

A novel, chemically-generated incisional negative pressure device performed effectively in clinical practice and was shown to be convenient and easy to use. Future studies to assess the effects on surgical site complications are warranted.

## Introduction

Negative pressure wound therapy (NPWT) is emerging as an extremely useful tool for the prevention of complications in closed surgical incisions. Multiple randomized studies have been reported and several meta-analyses show that, on balance, applying an average negative pressure of either -125 mmHg or -80 mmHg to the sutured or stapled incision in higher-risk patients and high-risk surgeries, can reliably reduce the frequency of surgical site infection (SSI) or surgical site complications (SSC) by around 40-50% [1,2].

The development of small, single-use, disposable electromechanical NPWT systems allowing patient mobility has significantly increased the number of randomized controlled trials (RCT) of closed incision NPWT (ciNPWT) that have been performed and the adoption of the therapy. Nevertheless, the relationship between the cost of currently available single-use NPWT systems (range US$250 - US$700) and the costs incurred for treating patients who suffer SSI or SSC must be balanced. For example, in an RCT in high BMI Danish C-section patients who had pre-gestational BMI>30, the utilization of single-use NPWT devices, costing around US$250, showed a statistically significant reduction in their wound complications (such as the need for post-operative antibiotics) [3]. Yet, from an economic standpoint, single-use NPWT was a statistically significant cost-saving option only in women at the highest risk of infection, namely those with a BMI>35 [4].

Thus, it is becoming clear that the number of patients and markets that will benefit from a prophylactic NPWT, may in part be determined by the cost of the devices. It follows, therefore, that new low-cost devices may be needed to substantially expand access to ciNPWT, particularly in low- and middle-income countries [5,6]. Additionally, improvements in ease of use, attaining and maintaining a seal, and reduction in the noise and vibration which disturbs sleep, are needs which remain unmet in existing electrically-powered, ciNPWT devices.

The purpose of the present investigation was to test the functionality of a novel, solid-state, non-electrical NPWT device for closed incisions for the first time in patients. This new device uses an exothermic chemical process to generate negative pressure at a clinically relevant average negative pressure of -80 mmHg over several days. This solid-state, closed incision, NPWT system can be manufactured at a much lower cost than the conventional electromechanical NPWT system, and does not generate medical electrical circuit board and battery waste for incineration.

## Materials and methods

Investigational device 

**Figure 1:**
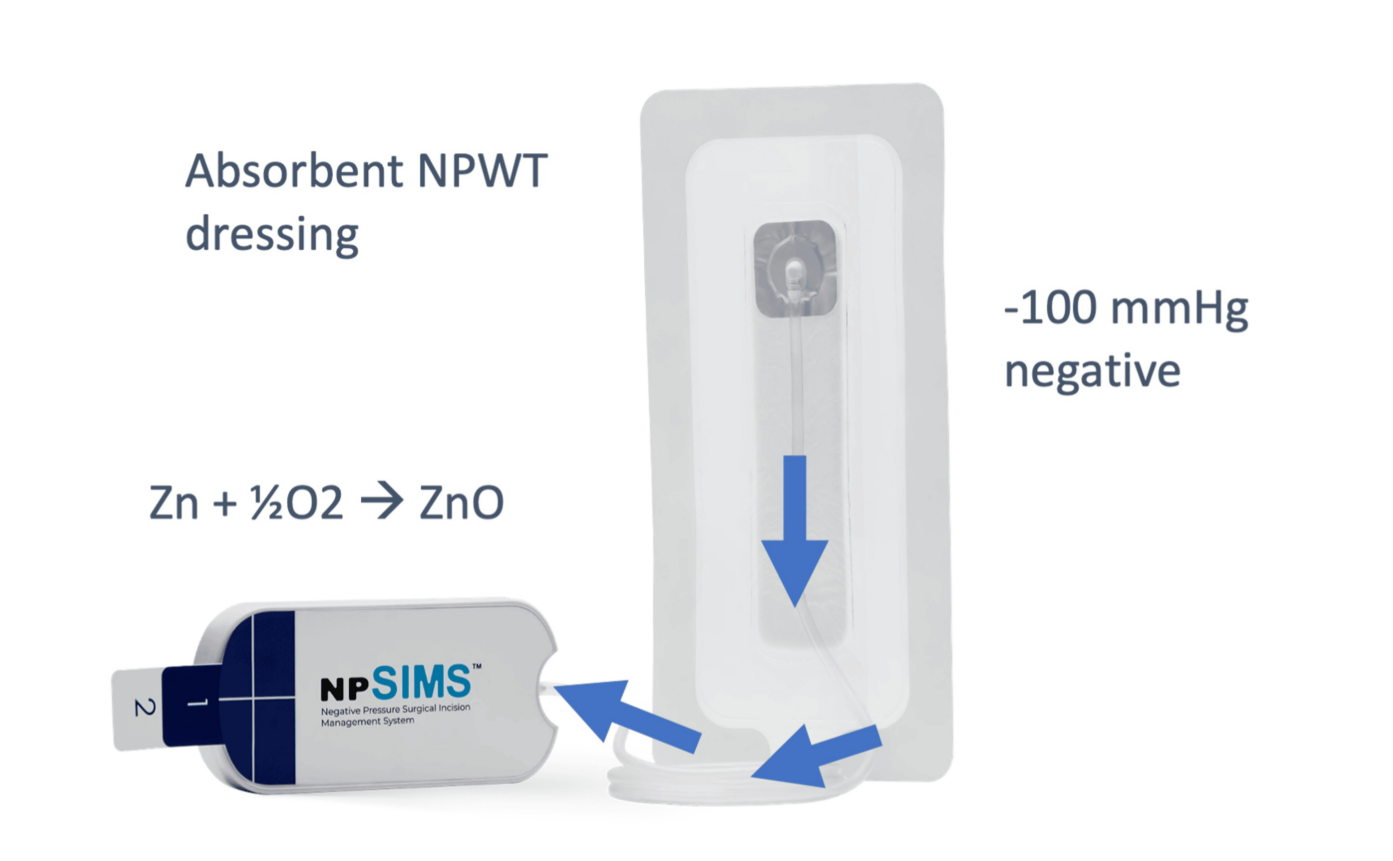
The negative pressure Surgical Incision Management System (npSIMS) The negative pressure Surgical Incision Management System (Aatru Medical, LLC, Ohio, USA) is a solid-state incisional negative pressure wound therapy (NPWT) system using chemical oxygen reduction technology to create and maintain a vacuum. Image used with the manufacturer's permission (Aatru Medical, LLC, Ohio, USA).

The solid state NPWT system (negative pressure Surgical Incision Management System or npSIMS) from Aatru Medical, LLC, Ohio, USA, consists of an adhesive dressing containing a super-absorbent material connected by tubing to a rigid, hollow vacuum chamber (Figure 1).

Within the vacuum chamber, there is a sealed package containing a mixture of zinc powder and catalyzed carbon powder wetted with a neutral pH, non-toxic electrolyte solution to allow the direct reaction of the zinc metal with oxygen in the air, according to the exothermic equation: Zn + ½O_2_ = ZnO. The packet is stable when sealed from the atmosphere and is activated with removal of a tear-off tab to expose access holes that admit air into the device.

**Figure 2:**
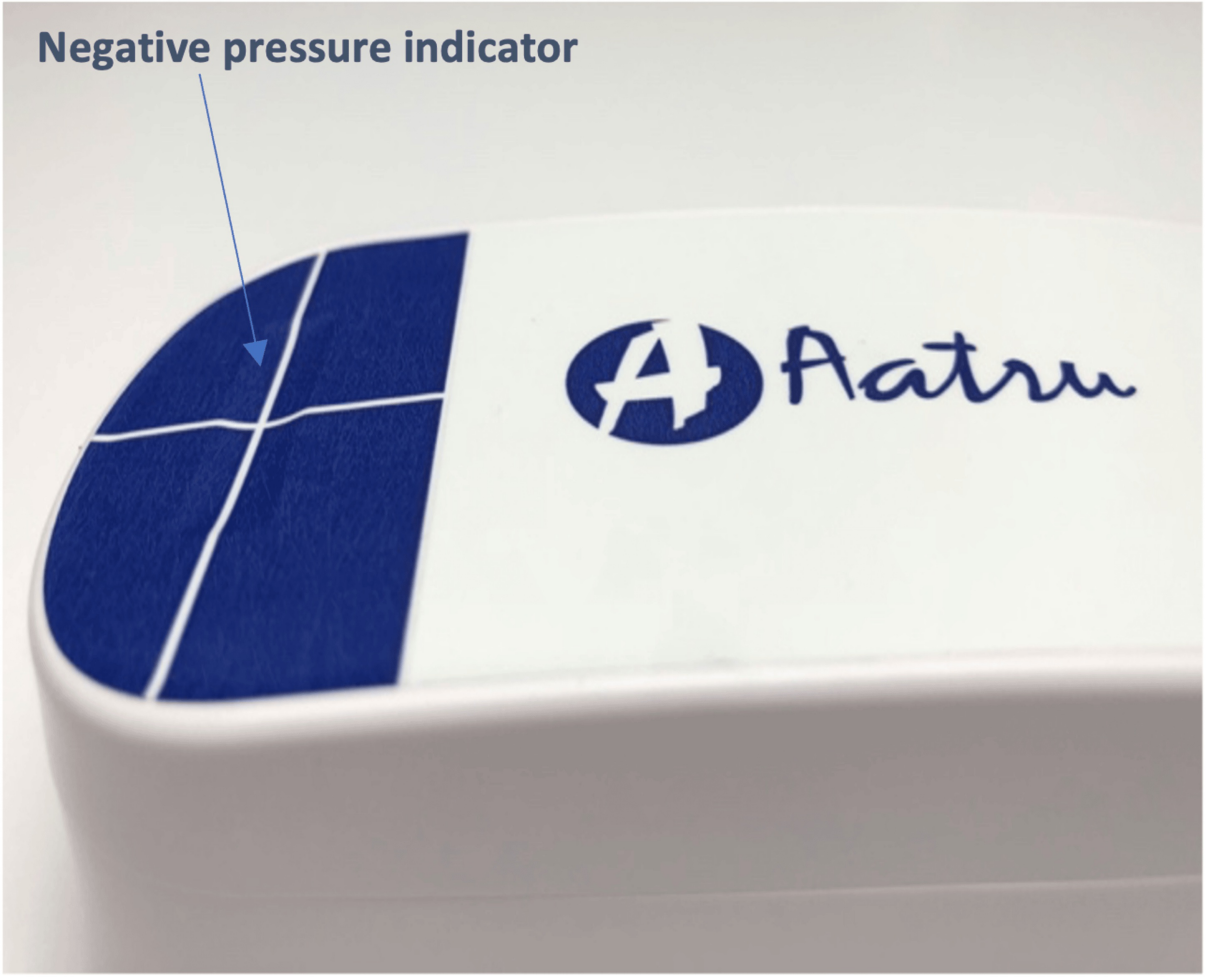
Negative pressure indicator Close up of the vacuum chamber with white crossed lines printed on an adhesive paper which is sucked into a grove on the upper surface of the vacuum chamber, indicating negative pressure in the system. Image used with the manufacturer's permission (Aatru Medical, LLC, Ohio, USA).

Oxygen represents 21% of the gas making up the atmospheric pressure of 760 mmHg at sea level. Thus, upon removal of oxygen, a maximum negative pressure (relative to the atmospheric pressure) of 0.21 x 760 mmHg = -159.6 mmHg could be created. In laboratory testing (not shown), an initial pressure of -110 mmHg is typically achieved. This is because not all the oxygen in the system is consumed, and the npSIMS's overall volume is reduced by the contraction of the absorbent dressing (under the forces of the reduced pressure). The vacuum holds steady with a gradual decline over a period of seven days to around -60 mmHg, as air diffuses through the dressing’s permeable outer membrane. An "all-or-none" mechanical pressure indicator (Figure 2) signifies that negative pressure has been activated in the device.

A novel silicone gasket seal minimizes the inward leakage of air at the boundary of the dressing. In tests conducted in a porcine incisional wound model, healing proceeded normally, as assessed by hematoxylin and eosin (H&E) histology in comparison with incisions covered for seven days with a conventional electromechanical NPWT system connected to the same npSIMS dressing [7]. The npSIMS is indicated for patients who would benefit from treatment with a NPWT device, as it may promote wound healing via removal of the exudate and infectious material from low-exuding wounds such as closed surgical incisions.

The npSIMS used in this study (Product Number: 700007-01), has dressings with dimensions of the absorptive pad as 14.4 x 3.6 cm. The width of the outer silicone gasket adhesive border is 2.2 cm and the width of the outer acrylic adhesive film border is 1.2 cm. The product used in the present study was sized sufficiently to manage incisions up to 13 cm in length. A minimum length of 5 cm was set for enrollment. The dimensions of the vacuum chamber (Figure 1) in the present study were 14.0 x 6.0 x 3 cm. The future plans of the manufacturer are to develop a larger selection of dressings to accommodate a wider range of incisional shapes and sizes. The vacuum chamber to dressing tubing is 80 cm in length, but can be cut-to-size to facilitate ease of handling before it is connected. 

Clinical study 

The title of the study was 'A Clinical Evaluation of a Novel, Single-Use, Negative Pressure Wound Therapy (NPWT) System for the Management of Closed Surgical Wounds.' The study was registered on ClinicalTrials.gov (NCT04488666) on July 27, 2020. Since the commencement of the study, the device has received 510(k) regulatory clearance in the USA (K201400) as a class II “non-powered suction apparatus device intended for negative pressure wound therapy."

The study was conducted at three sites in New Zealand. The first site was a plastic surgery center, the second site was an orthopedic unit, and the third site provided a cardiothoracic service. Initially, the success of the New Zealand authorities in minimizing the impact of the 2020/2021 SARS-CoV-2 pandemic on the healthcare system provided an unanticipated benefit that permitted the planning and implementation of the trial to proceed largely unhindered. However, travel into hospital clinics was kept to a minimum and as such, some assessments were restricted to those that could be reported by the participants during telephone assessments. Ethical approval was received from the New Zealand Ministry of Health, Health and Disability Ethics Committee (HDEC) reference number 20/STH/144 on November 9, 2020. The study design included a pre-planned pause in recruitment after five patients and scrutiny by an independent Data & Safety Monitoring Board (DSMB). Subsequently, prolonged SARS-CoV-2 restrictions in New Zealand resulted in a significant pause in recruitment before the study's completion. 

Study design

**Figure 3:**
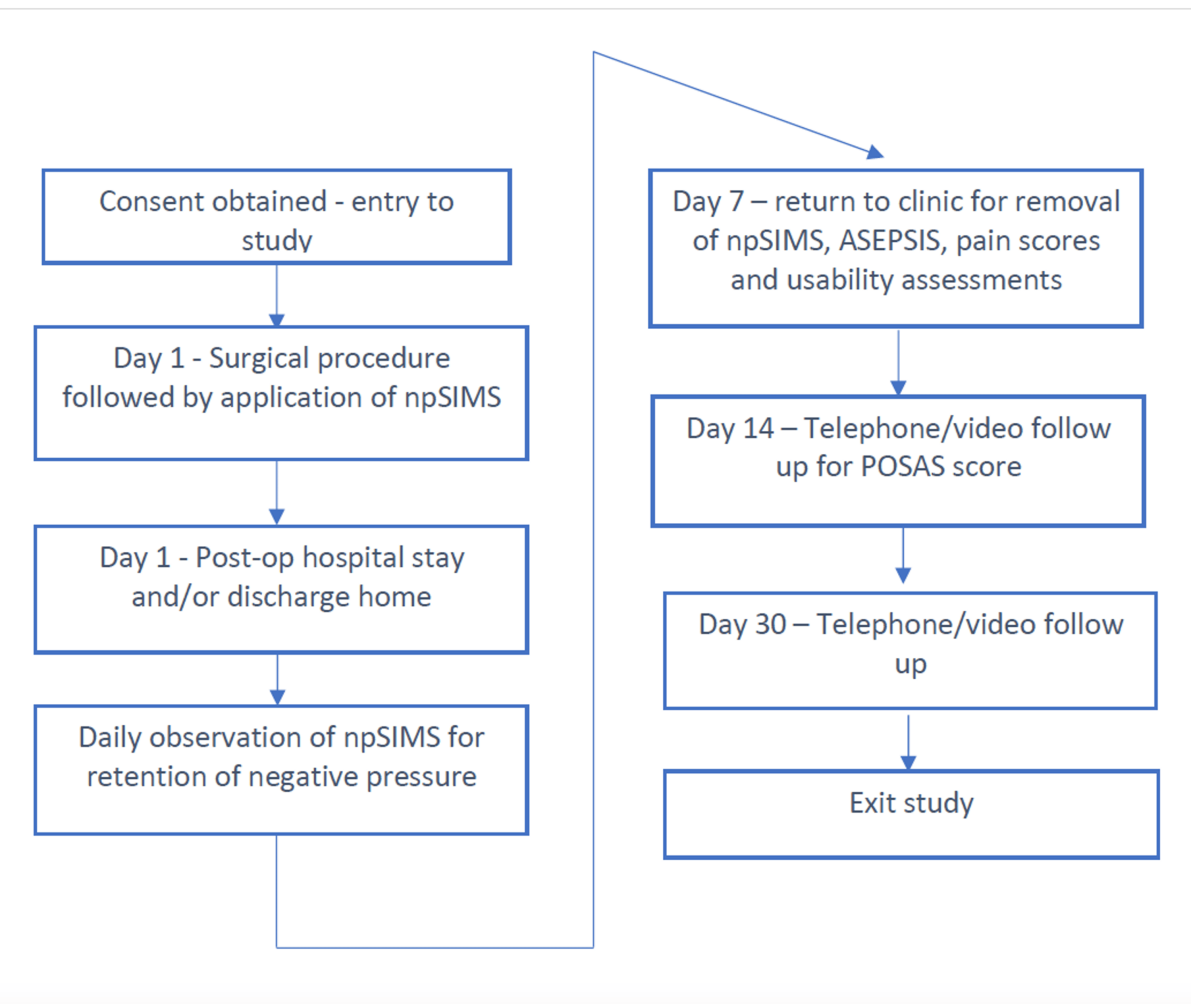
Study flow npSIMS: negative pressure Surgical Incision Management System; ASEPSIS: Additional treatment; Serous discharge; Erythema; Purulent exudate; Separation of deep tissues; Isolation of bacteria; and Stay as inpatient for a prolonged period (over 14 days); POSAS: Patient and Observer Scar Assessment Scale

The patient interactions with the study team are diagrammed in Figure 3. 

Following written consent, the subjects underwent their elective surgical procedure (day one). The wound was closed as normal and the investigational npSIMS device was applied and activated. A pain score was taken, and the patient was either discharged home or retained in the ward. Each subsequent day, the patient made a visual check of the device to verify if it was maintaining negative pressure. On day seven, the patients returned to the clinic for device removal and assessment of healing of the incision and surrounding skin with the Additional treatment; Serous discharge; Erythema; Purulent exudate; Separation of deep tissues; Isolation of bacteria; and Stay as inpatient for a prolonged period (over 14 days) or ASEPSIS tool [8,9]. An ASEPSIS score of 0-10 is designated as satisfactory healing; 11-20 a disturbance of healing; 21-30 a minor wound infection; 31-40 a moderate wound infection and >40 a severe wound infection. On Day 14, there was a telephone/video follow-up to assess the scar quality with the Patient and Observer Scar Assessment Scale (POSAS) tool [10]. On Day 30, there was a final telephone/video follow-up to check for any sign of SSI and to assess POSAS. The subject then exited the study.

Clinical assessments

The primary endpoint was the number of days of negative pressure delivered per incision. This assessment of negative pressure was made from the visual appearance of the dressing (firm and contracted) and the visual pressure indicator on the vacuum chamber which consisted of the white crossed lines printed on an adhesive paper which was sucked into a groove on the upper surface of the vacuum chamber, when the system was at negative pressure (Figure 2). Wound healing and the presence of SSI was assessed by the ASEPSIS validated tool which scores serous exudate, erythema, separation of deep tissues, and purulent exudate from a % of the length of the wound [8,9]. Scar quality was scored with the validated POSAS tool [10]. Pain (numerical ratings score or NRS) [11] assessments were also undertaken (Figure 3). Postoperative pain (day one) was assessed on an 11-point numerical rating scale with 0=no pain and 10=the worst pain you can imagine. 

Usability assessments

In addition to the clinical and technical assessments of the investigational system, the clinicians and subjects were also separately asked to score the ease of use of the device and its suitability for clinical practice. 

## Results

Patients

The first patient was recruited on January 26, 2021, and the last or 23rd patient exited the study on August 26, 2023. The demographic details of the participants are shown in Table 1.

**Table 1:** Patient demographics Numbers are mean±standard deviation (SD) and percent for categorical measures. Out of the 23 patients, 10 underwent cutaneous surgery for the removal of skin cancer and closure by sutures, another 10 underwent spinal surgery with a dorsal approach, and the last three patients underwent shoulder surgery, spinal surgery with an anterior approach, and video-assisted thoracoscopic surgery (VATS), respectively.

	Total enrolled (n=23)	Skin surgery (n=10)	Spinal surgery (n=10)
Age (years)	65.0±11.9	70.7±10.2	60.9±11.9
Age ≥65 years	12/23 (52.2%)	7/10 (70.0%)	4/10 (40.0%)
Female	10/23 (43.5%)	3/10 (30.0 %)	5/10 (50.0%)
Weight (kg)	91.1±20.4	83.7±19.7	99.3±19.5
Height (cm)	171.6±11.0	170.6±10.9	174.1±12.4
BMI (kg/m^2^)	30.9±6.8	28.3±3.9	33.2±8.1
Current smoker	1/23 (4.3%)	0/10 (0.0%)	1/10 (10.0%)
Type I diabetes	1/23 (4.3%)	0/10 (0.0%)	1/10 (10.0%)
Type II diabetes	8/23 (34.8%)	2/10 (20.0%)	5/10 (50.0%)
Steroid use	1/23 (4.3%)	0/10 (0.0%)	1/10 (10.0%)

Out of the study participants, 10 patients underwent cutaneous surgery for the removal of skin cancer, another 10 patients underwent spinal surgery with a dorsal approach, and the last three patients underwent shoulder surgery, spinal surgery with an anterior approach, and video-assisted thoracoscopic surgery (VATS), respectively. All the wounds were closed by sutures. Overall, the 23 participants had a mean age of 65.0±11.9 years and a mean BMI of 30.9±6.8 kg/m^2^ (Table 1). The patients in the plastic surgery group (n=10; central column in Table 1) were older and had a lower BMI than the patients in the spinal surgery group (n=10; right-hand side column in Table 1) i.e., 70.7±10.2 years vs 60.9±11.9 years and 28.3±3.9 vs 33.2 ± 8.1 kg/m^2^, respectively. Nine out of the 23 participants (39.1%) had diabetes. One had type I and eight patients had type II diabetes. Five of the patients with type II diabetes were in the spinal group. Only one patient was a current smoker (spinal surgery group). One patient undergoing spinal surgery used steroids for a chronic condition. A total of 18/23 (78.3%) participants were of the NZ-European race. Two patients were of Pacific ethnicity and the ethnicity of three patients was unknown.

Study completion

**Figure 4:**
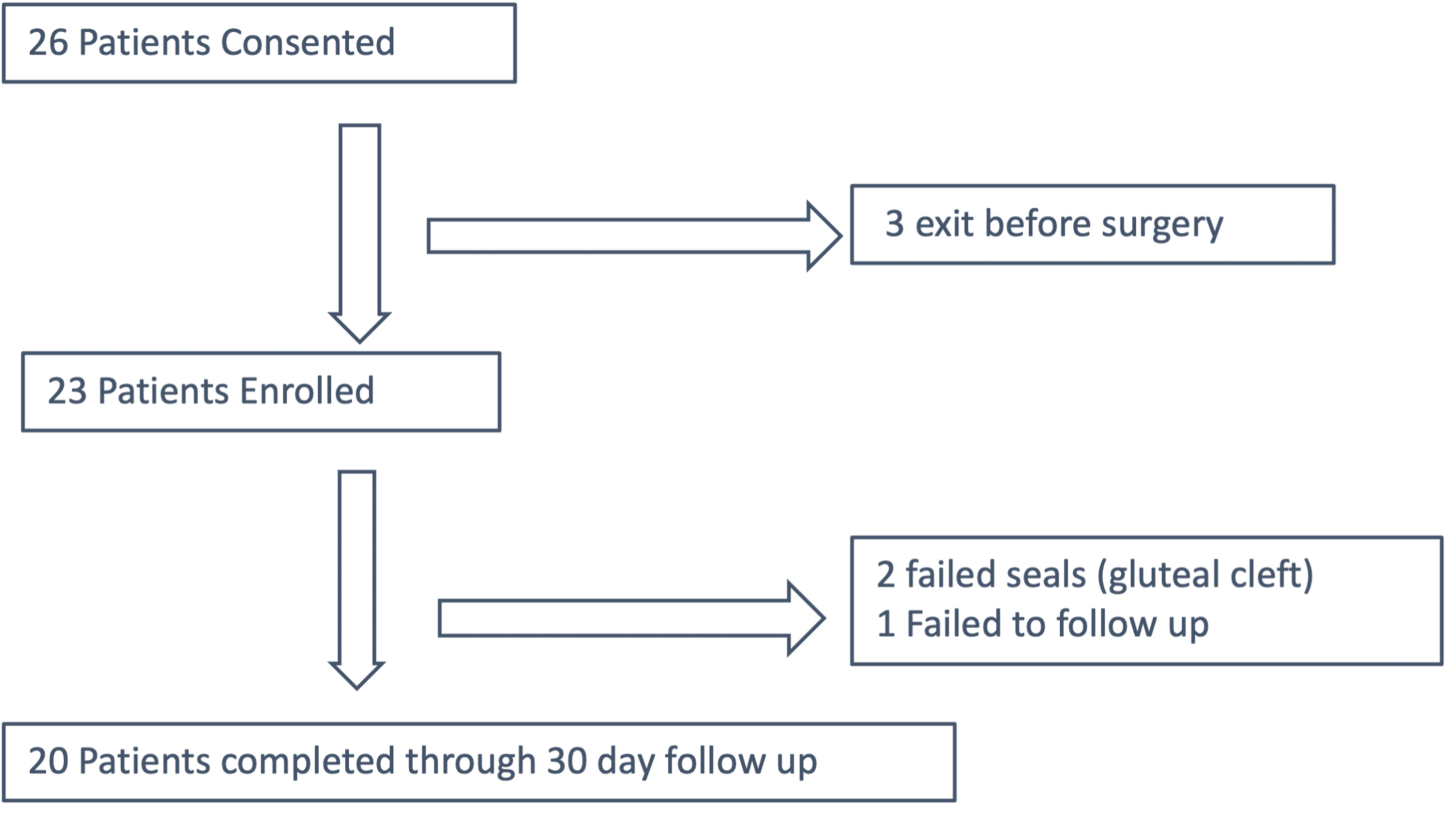
Summary of patient recruitment

Figure 4 summarizes patient recruitment and the number of patients completing the study. 

A total of 26 patients consented to the study but three exited the study before surgery leaving a total of 23 enrolled participants. Within the seven days following surgery, two patients with low lumbar incisions (spinal surgery) exited the study as it was not possible to make a satisfactory seal incorporating the gluteal cleft, and one patient was lost to the day 14 and day 30 follow up. These patients were included in the calculation of the mean number of days of negative pressure delivery per incision. Twenty patients completed the study through to the assessment on day 30. 

Surgical incisions

All patients had elective procedures. The types of surgery that could be included were C-section, cutaneous (skin) surgery, sternotomy and thoracotomy, shoulder, and spinal surgery. The details of the location and length of incisions are shown in Table 2. 

**Table 2:** Details of the incisions Numbers are mean±SD (N); †Video-assisted thoracoscopic surgery (VATS). Mean incision length (cm) for all: 7.6±2.6 (n=23); Plastic surgery: 6.0±1.5 (n=10); and Spinal surgery (dorsal incision): 8.4±2.5 (n=10). ^*^Enrolled patients were given a code reflecting the site they were recruited from e.g. 01, 02, 03 and the order of patients recruited at that site. Thus, 01-01 stands for the first patient recruited from the first site.

Participant*	Site location	Incision length (cm)	Reason for surgery
01-01	Upper back	9.0	Wide excision of lesion
01-02	Upper chest	6.0	Wide excision of lesion
01-03	Right shoulder	5.8	Wide excision of lesion
01-04	Right deltoid	4.8	Wide excision of lesion
01-05	Left forearm	6.0	Wide excision of lesion
01-07	Left upper chest	5.0	Melanoma - skin lesion removal
01-09	Back, upper right side	5.0	Melanoma - skin lesion removal
01-10	Right upper arm	5.0	Melanoma - skin lesion removal
01-11	Left shoulder	5.0	Melanoma - skin lesion removal
01-12	Right posterior neck	8.5	Melanoma - skin lesion removal
02-01	Posterior cervical spine	10.5	Fusion for non-union
02-02	Posterior lumbar spine	10	Revision instrumentation L4 to S1
02-03	Posterior lumbar spine	8	Spinal stenosis
02-04	Posterior lumbar spine	9	Spinal stenosis
02-05	Lumbar spine L5-S1	9	Decompression and fusion
02-06	Lumbar spine	13.0	Spinal stenosis and spondylolisthesis
02-07	Lumbar spine	5.5	Nerve root compression L5/S1
02-08	Lumbar spine	7.5	Lumbar disc prolapse
02-09	Left shoulder	11.5	Shoulder replacement for arthritis
02-10	Lumbar	4.5	L5/S1 Disc protrusion
02-12	Spinal surgery through anterior abdomen	12	L5/S1 Spondylosis
02-13	Lumbar - posterior	7	Spinal stenosis
03-01	VATS^†^ right lower lobectomy	6.5	Lesion, malignancy unknown

The overall mean incision length (n=23) was 7.6±2.6 cm. Amongst the 10 participants who underwent surgery for the removal of skin cancer, the mean incision length was 6.0±1.5 cm. In the 10 participants who had elective surgery to the spine, the mean incision length was 8.4±2.5 cm. As this study represented the first use of the new device in humans, the study protocol specified lower risk patients for the first five participants. Thus patients with lower risk of SSI or higher risk comorbidities such as BMI above 30 kg/m^2^ or diabetes, were recruited. There were no restrictions for the subsequent patients. Spinal patients are often considered higher risk for wound complications due to the difficulty of managing the post-operative wound when the patients lie on their backs. The surgeons placed a wound contact layer between the wound and the dressing a total of 9/23 (39%) times, mostly in the spinal surgery participants. None of the clinical teams elected to shorten the 80 cm tubing connecting the dressings to the vacuum chamber.

Maintenance of negative pressure

The primary objective of this study was to assess how many days of negative pressure could be delivered to the incision by the chemically-generated negative pressure device. If the device became non-functional or if exudate saturated the dressing, the device could be replaced, with the number of replacements being recorded. On day seven, 21/23 patients returned to the hospital clinic for scheduled wound and npSIMS device assessment; 20/21 npSIMS devices were in place and 20/20 were under negative pressure. Table 3 shows that the mean number of days of negative pressure delivery per incision was 6.3±1.6 (n=23) with a median of seven days. 

**Table 3:** Delivery of negative pressure by the npSIMS devices Numbers are mean±SD; median (minimum, maximum); npSIMS: negative pressure Surgical Incision Management System

Endpoints		Total (n=23)	Skin surgery (n=10)	Spinal surgery (n=10)
Days of negative pressure delivery per incision	mean	6.3±1.6	6.4±1.0	6.0±2.2
	median	7.0 (1.0, 9.0)	7.0 (4.0, 7.0)	6.5 (1.0, 9.0)
Number of devices per subject	mean	1.2±0.5	1.0±0.0	1.4±0.7
	median	1.0 (1.0, 3.0)	1.0 (1.0, 1.0)	1.0 (1.0, 3.0)
Days of negative pressure delivered per device	mean	5.9±1.9	6.4±1.0	5.3±2.3
	median	7.0 (0.3, 8.0)	7.0 (4.0, 7.0)	7.0 (0.3, 8.0)

**Figure 5:**
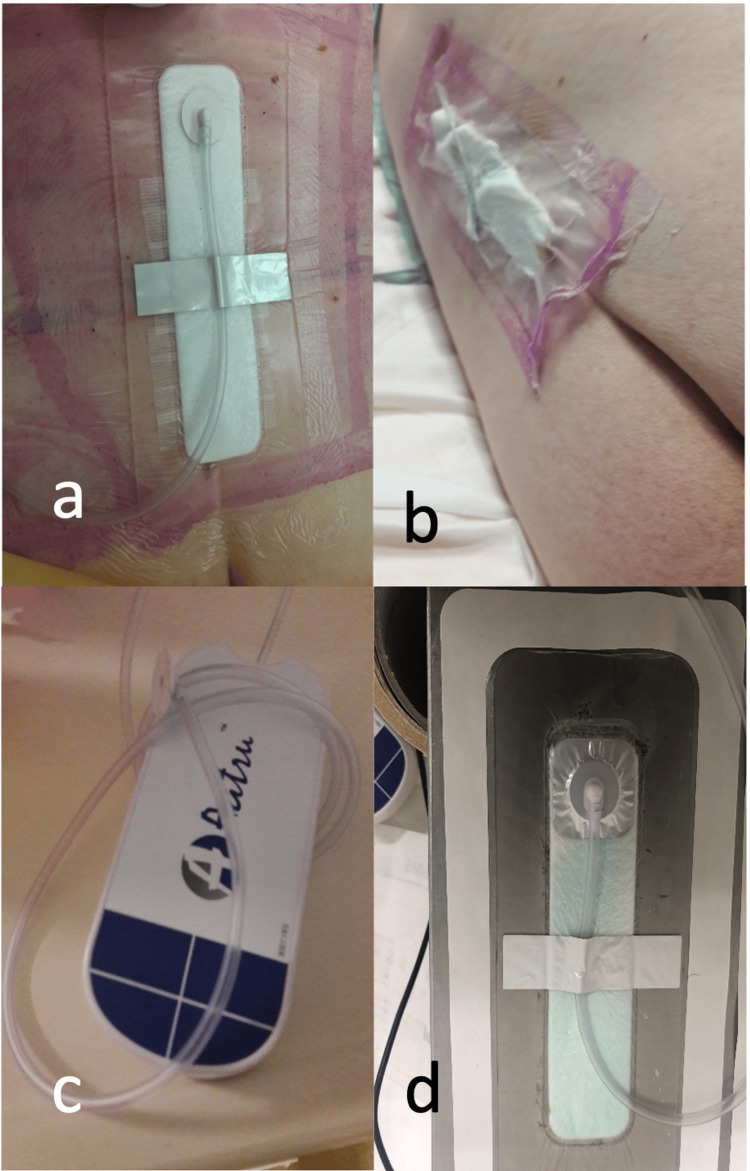
Device deficiencies and improvements The negative pressure Surgical Incision Management System (npSIMS) in patient 02-05 with lumbar spine L5-S1 decompression and fusion (9 cm incision).  (a) On day 2, npSIMS in place with a temporary solution, secondary tape applied to reduce shear force on the tubing. (b) On day 7, npSIMS dressing observed with zero negative pressure. (c) Recovered npSIMS device, port sheared off the dressing. (d) Device improvement with redesigned port-dressing adhesion. Image used with manufacturer's permission (Aatru Medical, LLC, Ohio, USA).

The mean number of devices per incision was 1.2±0.5. Overall, the mean number of days each device was able to operate was 5.9±1.9, although there was a wide range with a minimum of 0.3 days and a maximum of eight days. Table 3 reveals that on average more devices (1.4±0.7) were applied to spinal surgery wounds than plastic surgery incisions (1.0±0.0), suggesting that surgery-specific variables made it more difficult to treat spinal wounds than plastic surgery incisions. Loss of negative pressure during the seven-day postoperative period was captured as a device deficiency. There was one occasion where a patient with plastic surgery lost pressure after four days (at the shoulder location), and there were seven such occasions in the spinal surgery group. With incisions in the lower lumbar region, it seemed likely that loss of pressure was due to shear forces between the body and the dressing when the patient was lying in bed or sat in a chair. On four occasions (in three patients), the cause was the port and suction tubing getting detached from the dressing (Figures 5a-5c). 

One of these patients withdrew from the study after three attempts to get a seal. Clinicians attempted to mitigate this weakness through the application of an adhesive tape across the dressing to lower the risk of shear forces on the vacuum tubing (Figure 5a) This vulnerability was addressed with a design and manufacturing improvement made by the sponsor (Figure 5d). The improved design with greater adhesion between the port to the dressing was used by later participants in the study. On three other occasions, spinal surgery patients experienced a loss of negative pressure with no port detachment. 

Exudate management

Clinicians scored the level of exudate appearing in the npSIMS dressing at the follow-up visit on day seven on a five-point numerical rating scale. More than 80% of spinal patients had no exudate visible in the dressing whereas only 30% were assessed at that level for the plastic surgery incisions. In general, the incisions for plastic surgery displayed more exudate in the npSIMS dressings than those for spinal surgery at the time of dressing removal, although the larger number of devices used for the spinal patients maybe a partial explanation for this observation. No dressings were completely saturated with exudate by day seven. 

Pain assessment 

Postoperative pain (day one) was assessed on an 11-point numerical rating scale with 0=no pain and 10=the worst pain you can imagine. After the procedure and the application of the npSIMS devices, 10/23 (43.4%) patients reported no pain, with just 1/23 patients reporting a score of one. In practical terms, the use of postoperative pain medication in all but the patients with plastic surgery meant that study clinicians felt it was not possible to assess pain immediately after surgery. The pain scores were also reported at the follow-up on day seven. At this point 12/21 patients reported no pain; 4/21 scored pain at one, two patients scored pain at two, one scored three and two participants (both patients with spinal surgery) scored pain at seven. 

ASEPSIS on day seven

One of primary goals for the use of NPWT over closed incisions is to reduce the incidence of SSC such as SSI or dehiscence. The ASEPSIS score is a validated points-based system for evaluating a surgical wound so that gradations of the degree of infection are recorded, rather than a binary designation of infection or no infection [9]. Unfortunately, due to New Zealand's COVID-19 restrictions operating at the time, it was only possible to assess the ASEPSIS score on day seven as the rating required the clinician's assessment. A score of 0-10 is designated as satisfactory healing; 11-20 a disturbance of healing; 21-30 a minor wound infection; 31-40 a moderate wound infection and >40 a severe wound infection. A total of 15/21 wounds displayed no serous exudate from any point along the length of the wound; 5/21 showed exudate from less than 20% of the length of the wound (one point), and one subject had exudate from between 20 and 39% of the length of the wound (two points). In terms of erythema, 14/21 wounds showed no adjacent erythema; 5/21 had erythema of <20% of the wound length (one point), and two wounds scored two and three points respectively for erythema of 20-39% and 40-60% of the length of the incision. Overall, all wounds scored between 0-10, indicating normal wound healing in 21/21 participants on the seventh day.

Skin blisters on day seven

With other incisional NPWT devices, a previously described adverse event has been the development of blisters on the surrounding skin beneath the adhesive film dressing [12]. Thus, an assessment for skin blisters was made on day seven. Two subjects (2/21, 9.5%), who had undergone spinal surgery, displayed skin blistering.

POSAS scores on day 14 and day 30

Scar quality was measured by the POSAS [10]. Again, because of the COVID-19 restrictions in New Zealand at the time, the patients did not return to the clinic on day 14 or day 30. So, only the patient element of the POSAS score could be recorded. A total of 20 patients were included in the day 14 and day 30 scar assessments. The mean/median scores were all between 1 and 2.5 indicating that, in general, the incisions were not painful, few had symptoms of itch, and were of normal color, texture and appearance. However, one individual scored aspects of their scar at seven and eight points.

Ease of use assessment by participants on day seven

An important aspect of the study was to gain an understanding of how easy it was for the patient to manage recovery and return to normal activities in the postoperative period whilst using the npSIMS device. Table 4 shows the participants' responses (n=21) to questions on day seven, just after they had finished wearing the device, about bathing, dressing, and sleeping (scale of 1 to 4) and overall ease of wearing the device and comfort (scale of 1 to 5).

**Table 4:** Ease of use for the participant (on day seven) Scores were made over the following ranges: Bathing - Range from 1 (without any difficulty), 2 (with some difficulty), 3 (with much difficulty), and 4 (unable to do); Getting dressed - Range from 1 (without any difficulty), 2 (with some difficulty), 3 (with much difficulty), and 4 (unable to do); Sleeping - Range from 1 (without any difficulty), 2 (with some difficulty), 3 (with much difficulty), and 4 (unable to do); Overall assessment of wearing the device - Range from 1 (very easy) to 5 (very difficult); Overall, how comfortable was wearing the device during the last week? Range from 1 (very comfortable) to 5 (very uncomfortable). Numbers are mean±SD (number of participants); median (minimum, maximum) for continuous measures, and percentage for categorical measures.

Variable	Mean score*	Median score*
Bathing	1.7±0.7 (n=21)	2.0 (1.0, 3.0)
Getting dressed	1.6±0.5 (n=20)	2.0 (1.0, 2.0)
Sleeping	1.6±0.7 (n=21)	1.0 (1.0, 3.0)
Overall assessment of wearing the device	1.9±1.2 (n=21)	1.0 (1.0, 5.0)
Overall, how comfortable was wearing the device during the last week?	1.8±1.2 (n=21)	1.0 (1.0, 5.0)
Over the last week have you had any issues related to the device?	7/21 (33.3%)	

Generally, participants experienced some difficulties with daily activities such as bathing and dressing (mean scores of 1.7 and 1.6), although some difficulty might be expected in any case with a surgical incision and a dressing. The mean score for sleeping was 1.6 (median score of one), indicating little difficulty in sleeping. For overall wearing and comfort, mean scores were 1.9 and 1.8 respectively on the five-point scale, although individual patients with difficulty in maintaining the seal scored five, indicating difficulty managing the device. Comments from several study participants were captured, concerning how to manage the vacuum chamber more easily. One of the suggestions was a clip of some kind to fix the vacuum chamber to the patient's clothes.

Clinicians' ease of use and suitability assessments

On day seven of the follow up, the clinicians were asked to rate the ease of use of the device on a five-point scale with one being high and five being low. The mean score across the 21 evaluable patients was 1.1±0.3 with a median and range of 1.0 (1.0, 2.0) respectively. On day 14, the participant was followed up by telephone or video, and the clinician was asked to rate the overall acceptability of the new npSIMS, on a five-point scale with one being high and five being low. The score was unanimously 1.0 (n=16) out of the ratings that were recorded, suggesting that clinicians felt it was an acceptable solution to the needs of the postoperative patient.

Adverse events

As this was a first-in-human study, close attention was paid to the detection and recording of adverse events. There were a total of nine adverse events recorded; two in two patients from the plastic surgery group and seven in three patients from the spinal surgery group. Table 5 shows that in the plastic surgery group, two subjects (participants 01-09 and 01-12) reported SSIs during the telephonic follow-up on day 14.

**Table 5:** Adverse events *Enrolled patients were given a code reflecting the site they were recruited from e.g. 01, 02, 03 and the order of patients recruited at that site. Thus, 01-01 stands for the first patient recruited from the first site. A total of nine adverse events were recorded; two in two plastic surgery participants and seven in three spinal surgery participants.

Participant*	Details of the incision	Details of the adverse event(s)
01-09	Plastic surgery for melanoma removal from the back, upper right side; horizontal incision: 5.0 cm, BMI 31.9 kg/m^2^, Type II diabetes	1 x non-device related mild adverse event: Surgical Site Infection - redness and swelling at the site noted by the general practitioner (GP) on post-op day 10 when sutures were removed – no temperature or pain. GP prescribed oral antibiotics, reported as resolved by post-op day 28
01-12	Plastic surgery for melanoma removal from the right posterior neck; horizontal incision: 8.5 cm, BMI 30.19 kg/m^2^	1 x non-device related mild adverse event: Surgical Site Infection - redness and swelling at the site noted by GP on post-op day 9 when sutures were removed – no temperature or pain. GP prescribed oral antibiotics, reported as resolved by post-op day 42
02-06	Spinal surgery for lumbar spinal stenosis and spondylolisthesis; incision: 13 cm	3 x non-device related mild adverse events arising on day 1 (Itching), day 3 (skin blisters), day 3 (skin irritation)
02-07	Spinal surgery for nerve root compression L5/S1; incision: 5.5 cm	1 x non-device related mild adverse event on day 5 (COVID-19 positive); 1 x non-device related moderate serious adverse event on day 11 (wound dehiscence) resolved on day 37 & 38, respectively
02-08	Spinal surgery for lumbar disc prolapse; incision: 7.5 cm	2 x device-related mild adverse events on day 6 (Itching) and day 7 (skin blisters), resolved on day 14

Redness and swelling at the incision site had been observed by their general practitioners (GPs) when the patients visited them for suture removal on days 10 and nine, respectively. Both were treated with oral antibiotics. Each case was captured as a mild, non-device related adverse event which resolved successfully. In the spinal surgery patients, subject 02-06 experienced three adverse events of skin irritation, itching, and skin blisters. The clinician categorized these as non-device related mild adverse events. In subject 02-08, itching on day six and skin blisters on day seven were designated by the clinician as mild device-related adverse events. These had resolved by day 14. The spinal surgery subject 02-07 suffered two adverse events. The patient became COVID-19 positive on day five (mild non-device related adverse event), and suffered from surgical wound dehiscence on day 11, which necessitated surgical wash-out and re-suturing (moderate serious non-device related event). The adverse event had resolved by day 38. 

## Discussion

Negative pressure devices are increasingly deployed for surface management of closed incisions [1,2]. NPWT provides for an overall simplification of wound management. It decreases tension at the skin edge, controls edema, and manages serous exudate [2]. A novel, non-electrical method for generating a vacuum has been incorporated into a single-use NPWT device, which was evaluated in a first-in-human study in 23 patients. The source of the vacuum was provided by an oxygen-scavenging chemical reaction, which maintained negative pressure following a single activation for a mean of 5.9±1.9 days in 23 patients on a wide range of incisions at different anatomical locations. 

At first glance, reducing the level of oxygen on the surface of a healing wound under NPWT might be viewed as counterproductive. However, recent studies have shown that conventional NPWT performed by the bedside or by single-use electromechanical pumps ordinarily creates a degree of reduced oxygen partial pressure. Biermann et al. have shown that oxygen levels drop by 2.275% for every 25 mmHg of negative pressure [13]. As discussed by Biermann et al., changes in the levels of oxygen at the NPWT wound surface might be linked with changes in the levels of different types of bacteria in the wound, a finding that has previously been documented in clinical studies of NPWT [14]. Oxygen is important for numerous aspects of tissue regeneration; however, the trigger for many wound healing processes, such as angiogenesis - the growth of new blood vessels - is the detection of partial hypoxia by the tissue [15]. In reality, it is probably the oxygen delivered to the wounds via blood flow and not atmospheric oxygen outside the wound bed that meaningfully contributes to wound healing. The use of an oxygen scavenger technology to create a solid-state negative pressure device may simply be an extension of one of the mechanisms of action of existing NPWT. Either way, preliminary studies (data not shown) suggest that the level of reduced oxygen in NPWT dressings is very similar in chemically-generated negative pressure and conventional electromechanical NPWT devices.

In this study, two of the patients undergoing plastic surgery for skin cancer removal were reported as having SSI by community GPs who observed redness and swelling at the incision site on days nine and 10. The patients were treated with oral antibiotics. Notably, on the day seven face-to-face follow-up with the hospital-based clinicians, no hints of infection, exudate or pain were recorded using the ASEPSIS wound assessment tool, which allows for some redness and swelling in a normally healing wound [8,9]. It is not possible to know whether the clinicians would have scored these wounds as an SSI if they had seen them two to three days later, though there could be a difference in the community assessment by a specialist vs a non-specialist. 

In the spinal surgery group, 1/10 patients suffered an unequivocal wound infection and dehiscence. In a recent metanalysis of studies, including 1036 patients undergoing spinal decompression and/or fusion, treated with either standard care or electromechanical closed incision NPWT, Lambrechts et al. [16] reported standard care and ciNPWT SSI frequencies of 11.3% vs 4.5% (P=0.0103) and dehisced wound complications of 7.5% vs 4.6% (p=0.232), respectively. It would not be unexpected, therefore, for one out of 10 subjects in the present study to suffer a wound SSI or dehiscence. As this was not a comparative study, it is impossible to judge the efficacy of the study device in minimizing such complications.

Two participants who had undergone spinal surgery displayed some skin blistering in the peri wound area (2/21=9.5%). Skin blistering has been observed in previous incisional NPWT studies. In large study of high BMI C-section patients, 7.0% of patients treated with incisional NPWT suffered skin blisters [12]. The frequency in this study is, therefore, typical of that found in previous studies. With just two occurrences of skin blistering, it is not possible to be sure what the true incidence would be in a larger population of patients with this technology. Training in application techniques which avoid applying adhesive film on incisional NPWT dressings under tension has been shown to minimize the issue [17].

Limitations

The prevailing limitation in this study was the need to reduce face-to-face follow-up assessments due to COVID-19. This meant that the ASEPSIS tool was only applied by clinicians during face-to-face assessments on day seven. It would have been preferable to have used the ASEPSIS tool on day 14 and day 30 as well.

## Conclusions

This clinical study has shown that a novel, solid-state, ciNPWT device, in which a vacuum is provided by an oxygen-consuming chemical reaction, can generate and maintain negative pressure and manage the exudate from closed incisions for a mean of 6.3 days. Study clinicians familiar with currently available electromechanical single-use NPWT devices welcomed the npSIMS devices, which were readily applied, convenient and easy to use. The silent operation and the ability to get an airtight seal were useful features for clinical practice.

A number of further studies could be envisaged. Future work with a greater range of dressing shapes and sizes would be valuable, so that experience with different types of surgical incisions could be obtained. Larger dressing sizes with greater margins around the wound might reduce incidents of pressure loss, such as was seen in some of the spinal surgery cases. Finally, larger randomised comparative studies would be essential to determine whether the application of the npSIMS system can reduce the incidence of SSI across a range of wounds.
